# The KNee OsteoArthritis Prediction (KNOAP2020) challenge: An image analysis challenge to predict incident symptomatic radiographic knee osteoarthritis from MRI and X-ray images

**DOI:** 10.1016/j.joca.2022.10.001

**Published:** 2022-10-12

**Authors:** J. Hirvasniemi, J. Runhaar, R.A. van der Heijden, M. Zokaeinikoo, M. Yang, X. Li, J. Tan, H.R. Rajamohan, Y. Zhou, C.M. Deniz, F. Caliva, C. Iriondo, J.J. Lee, F. Liu, A.M. Martinez, N. Namiri, V. Pedoia, E. Panfilov, N. Bayramoglu, H.H. Nguyen, M.T. Nieminen, S. Saarakkala, A. Tiulpin, E. Lin, A. Li, V. Li, E.B. Dam, A.S. Chaudhari, R. Kijowski, S. Bierma-Zeinstra, E.H.G. Oei, S. Klein

**Affiliations:** † Department of Radiology # Nuclear Medicine, Erasmus MC University Medical Center, Rotterdam, the Netherlands; ‡ Department of General Practice, Erasmus MC University Medical Center, Rotterdam, the Netherlands; § Department of Biomedical Engineering, Cleveland Clinic, Cleveland, USA; ‖ Department of Radiology, New York University Langone Health, New York, USA; ¶ Department of Radiology, University of California, San Francisco, San Francisco, USA; # Research Unit of Medical Imaging, Physics and Technology, University of Oulu, Oulu, Finland; †† Department of Diagnostic Radiology, Oulu University Hospital, Oulu, Finland; ‡‡ Akousist Co., Ltd., Taoyuan City, Taiwan; §§ Department of Computer Science, University of Copenhagen, Copenhagen, Denmark; ‖‖ Department of Radiology, Stanford University, Stanford, USA; ¶¶ Department of Orthopedics # Sport Medicine, Erasmus MC University Medical Center, Rotterdam, the Netherlands

**Keywords:** Deep learning, Knee osteoarthritis, Machine learning, Magnetic resonance imaging, Prediction, Radiography

## Abstract

**Objectives:**

The KNee OsteoArthritis Prediction (KNOAP2020) challenge was organized to objectively compare methods for the prediction of incident symptomatic radiographic knee osteoarthritis within 78 months on a test set with blinded ground truth.

**Design:**

The challenge participants were free to use any available data sources to train their models. A test set of 423 knees from the Prevention of Knee Osteoarthritis in Overweight Females (PROOF) study consisting of magnetic resonance imaging (MRI) and X-ray image data along with clinical risk factors at baseline was made available to all challenge participants. The ground truth outcomes, i.e., which knees developed incident symptomatic radiographic knee osteoarthritis (according to the combined ACR criteria) within 78 months, were not provided to the participants. To assess the performance of the submitted models, we used the area under the receiver operating characteristic curve (ROCAUC) and balanced accuracy (BACC).

**Results:**

Seven teams submitted 23 entries in total. A majority of the algorithms were trained on data from the Osteoarthritis Initiative. The model with the highest ROCAUC (0.64 (95% confidence interval (CI): 0.57–0.70)) used deep learning to extract information from X-ray images combined with clinical variables. The model with the highest BACC (0.59 (95% CI: 0.52–0.65)) ensembled three different models that used automatically extracted X-ray and MRI features along with clinical variables.

**Conclusion:**

The KNOAP2020 challenge established a benchmark for predicting incident symptomatic radiographic knee osteoarthritis. Accurate prediction of incident symptomatic radiographic knee osteoarthritis is a complex and still unsolved problem requiring additional investigation.

## Introduction

Osteoarthritis (OA) is the most common joint disease which affects over 250 million people worldwide^1^. OA is a leading cause of disability and results in a tremendous burden for patients and society^2^. At the end stage of the disease, total knee replacement (TKR) surgery is the only available treatment option. However, during the early stages of OA, the disease might be more amenable to modification^3,4^. Thus, there is an important need to identify subjects at high risk of knee OA incidence to prevent or slow down the disease process.

In addition to known clinical risk factors for knee OA, imaging may help to identify knees at high risk for OA incidence^5–9^. Machine learning approaches have been proposed to enhance the analysis of large imaging datasets^10^ and have shown promising results for prediction of OA incidence^7,9,11^. Deep learning is an advanced machine learning method that can automatically extract relevant image features using convolutional neural networks (CNN) and has previously been applied for prediction of onset and progression of OA^12–15^. These studies include prediction of incidence and progression of radiographic knee OA from X-ray images using a modified ResNet^14,16^, prediction of progression of radiographic medial joint space loss from X-ray images using a DenseNet^12,17^, and prediction of the likelihood of a patient undergoing TKR from X-ray images using a pre-trained ResNet^13,18^ and from magnetic resonance imaging (MRI) data using a DenseNet^15^.

Typically, such prediction models are optimized, often by accident, for specific imaging datasets and it is unclear how different methods would perform on previously unseen data from different sources. Furthermore, direct comparison of the methods is difficult due to the different datasets and data partitions. To enable better comparison of methods, the concept of “grand challenges” has emerged in the medical image analysis research community and has been successfully applied to many specific image analysis and prediction tasks. These challenges aim to assess the performance of multiple different methods on the same data, using the same evaluation protocol, where the participants typically do not have access to the ground truth and hence cannot overfit their models^19,20^. Previous OA-related challenges include the Segmentation of Knee Images 2010 (SKI10) challenge^21^, the 2019 International Workshop on Osteoarthritis Imaging (IWOAI) knee MRI segmentation challenge^22^, and the MRNet challenge for automated interpretation of diagnostic knee MRI^23^, but a challenge on the prediction of OA has not been presented to date.

In this work, we describe the methodology and present the results from the KNee OsteoArthritis Prediction (KNOAP2020) challenge. The aim of this challenge was to objectively compare different methods for the prediction of incident symptomatic radiographic knee OA (according to the combined American College of Rheumatology (ACR) criteria^24^) within 78 months on a test set with blinded ground truth. We provided a test set (MRI and X-ray image data along with clinical risk factors at baseline) of 423 knees without symptomatic radiographic knee OA at baseline and the task was to identify which knees developed incident symptomatic radiographic knee OA within the follow-up period.

## Methods

### Data

Data for this study originated from the Prevention of Knee Osteoarthritis in Overweight Females (PROOF) study (ISRCTN 42823086)^25^. The PROOF study is a preventive randomized controlled trial that included 407 middle-aged, overweight/obese (body mass index (BMI) ≥ 27 kg/m^2^) women at baseline. The Medical Ethics Committee of Erasmus MC University Medical Center approved the PROOF study and all study participants gave written informed consent. For this challenge, we selected 453 knees (242 individuals) without symptomatic radiographic knee OA (combined clinical and radiographic ACR criteria^24^) at baseline and that had baseline X-ray and MR images and follow-up data at 2.5 years and/or 6.5 years for defining incident symptomatic radiographic knee OA. Knees with Kellgren–Lawrence (KL) grade^26^ > 1 at baseline were excluded. Furthermore, participants who dropped out from the study before the last follow-up timepoint and had not developed symptomatic radiographic knee OA at the previous timepoints were excluded.

### Challenge design

The data were split into a small training dataset (30 knees) and test set (423 knees) and were shared through the grand-challenge website (https://knoap2020.grand-challenge.org). The training data was meant for fine-tuning and contained background variables, clinical risk factors, X-ray and MR images, and outcome labels. The test set contained the same data except the outcome label, i.e., the participants did not know the actual outcome of each knee in the test set. An open invitation was sent to research teams worldwide to participate in the challenge. Participants were required to sign a data use agreement before downloading the data. Each participant was allowed to submit maximum of five submissions. Each submission was required to include the probability of each knee to develop incident symptomatic radiographic knee OA within the follow-up and a short description of the algorithm. The submissions were submitted via the challenge website. For comparison, one team provided a reference submission using only MRI data and one team provided four reference submissions using only clinical variables (Table I and Supplementary Material) and these submissions were not ranked. The test set of the challenge was released in August 2020, the submission system was opened in October 2020, the deadline for the submissions was in January 2021, and the results were presented at the IWOAI2021 workshop^27^ in July 2021.

### Imaging data

The imaging data of the challenge consisted of knee X-ray and MR images. The images were converted to the NIfTI file format (https://nifti.nimh.nih.gov)^28^ and were stored and shared via the Health-RI XNAT platform (https://www.health-ri.nl/services/xnat)^29^. The X-ray data consisted of semi-flexed posterior-anterior knee radiographs that were taken according to the metatarsophalangeal protocol^30^. The X-ray image data were acquired with multiple devices and protocols. X-ray images with a Swissray (ddR Compact System, Hochdorf, Switzerland) radiography system were acquired with 60 kVp and 10 mAs and the pixel size was 0.104 mm × 0.104 mm. X-ray images with General Electric (GE) (Thunder Platform, Waukesha, USA) radiography systems were acquired with 60–70 kVp and 3–5 mAs and the pixel size varied from 0.190 mm × 0.190 mm to 0.192 mm × 0.192 mm. Information about the X-ray device manufacturer, tube voltage, exposure, and pixel size were available for the participants.

The challenge MRI data were acquired with three different scanners (1.0T Philips Intera, Eindhoven, The Netherlands; 1.5T Siemens Symphony, Erlangen, Germany; and 1.5T Siemens Magnetom Essenza, Erlangen, Germany) and contained a coronal 2D proton density (PD) weighted sequence and a sagittal 3D sequence with water excitation (Supplementary Table 1). The scanner manufacturer, repetition time, echo time, flip angle, slice thickness and spacing, and voxel size were available for the participants.

### Clinical covariables

Clinical covariables for the KNOAP challenge were shared with the participants through the challenge website. The following variables were provided^25,31^: participant identification number, age, BMI, side (left/right), baseline KL grade (0/1)^26^, history of knee injury, presence of mild symptoms, varus malalignment, presence of Heberden nodes, joint line tenderness, crepitus, morning stiffness, and postmenopausal status.

Injury was defined as whether or not the women had ever visited a doctor for knee injury (no/yes). Mild symptoms were assessed with the question “Did you experience any pain in or around your knee within the past 12 months?” (no/yes). Both hands of the individuals were examined for Heberden’s nodes (no/yes). Morning stiffness was evaluated with the Knee injury and Osteoarthritis Outcome Score (KOOS) subscale on stiffness^32^ and it was defined as being present when the knee had moderate/much/very much joint stiffness after sleeping (versus no/little joint stiffness). Both knees of the individuals were examined for pain at palpation of the medial and lateral joint line (no/yes) and tested for crepitus during active flexion and extension of the knee (no/yes). Postmenopausal status was defined after 12 consecutive months of amenorrhea.

### Outcome measure

Incident symptomatic radiographic knee OA according to the combined clinical and radiographic ACR criteria^24^ was the binary outcome variable in this challenge. Symptomatic knee OA was defined as knee pain and a definite tibiofemoral osteophyte of any size in the same knee^25^. Knee pain was assessed with the question “Did you experience pain in or around left, right, or both knees during most days in the past month?”. Incident symptomatic radiographic knee OA was defined as the presence of symptomatic radiographic knee OA at 2.5 and/or 6.5 years follow-up that was not present at baseline.

### Training data

We provided a training dataset of 30 knees with the outcome variable available for the participants, to allow them to finetune their models on representative data. In addition, the participants were free to use any other source of training data. We anticipated participants using the Osteoarthritis Initiative (OAI) data for this purpose, since it is publicly available, has a long follow-up, and includes both knee X-ray images and 3T MRI scans. The OAI is a longitudinal multi-center study that includes clinical and imaging data over a 9-year follow-up period in 4,796 subjects (45–79 years old) at risk of knee OA. Details of the OAI data collection and study design have been previously reported^33^. The OAI MRI protocol includes sagittal 3D dual-echo in steady state with selective water excitation (DESS WE) and coronal 2D intermediate-weighted turbo spin-echo (TSE IW) sequences that resemble the MRI sequences in the KNOAP challenge test data. For convenience of the participants, we provided a variable defining incident symptomatic radiographic knee OA within 72 months for all baseline subjects in the OAI data. We also proposed a randomly selected test set of 108 knees from the OAI with characteristics similar to the knees in the KNOAP challenge test set (the same age and BMI ranges and sex), enabling participants to validate the performance of their models in the OAI data and enabling a direct comparison of training results between different models.

### Statistical analyses

To assess the performance of the submitted models, we used the area under the receiver operating characteristic curve (ROC AUC) and balanced accuracy (BACC). ROC AUC was used as a primary measure to rank the submissions, whereas BACC was used as secondary measure and this information was available for the participants before they participated in the challenge. Due to the class imbalance, post–challenge analyses included calculation of the area under the precision–recall curve (PR AUC) values^34^ as well as sensitivities and specificities of the submissions. We calculated 95% confidence intervals (CIs) by bootstrapping the test set 1,000 times. Python (v. 3.7.2) and Scikit-learn (v. 0.23.1)^35^ library were used for calculation of the metrics. The statistical significance of the difference between the models was assessed using DeLong’s test^36^.

## Results

### Dataset characteristics

In the training set and test set, 5/30 (16.7%) and 70/423 (16.5%) knees developed incident symptomatic radiographic knee OA within the follow-up, respectively. Supplementary Table 2 shows the distribution of knees between different scanners used to acquire the study data. At baseline, the mean age and BMI were 56.0 (standard deviation (SD): 2.8) years and 32.4 (SD: 3.7) kg/m^2^ in the training set, respectively, and 55.7 (SD: 3.2) years and 31.7 (SD: 3.7) kg/m^2^ in the test set, respectively.

### Algorithms

Of the 15 teams that registered to the challenge, seven teams provided altogether 23 submissions (Table I and Supplementary Material). Of these teams and submissions, one team provided a reference submission using only MRI data (*UC-MRI*) and one team provided four reference submissions using only clinical variables (*EMC-1*, *EMC-2, EMC-3, EMC-4*). The majority of the submissions used deep learning for extracting information from the images. All algorithms, except *UC-MRI*, were trained using knees from the OAI database. *UC-MRI* algorithm was trained on the KNOAP training set of 30 knees.

### Overall results

**Fig. 1:**
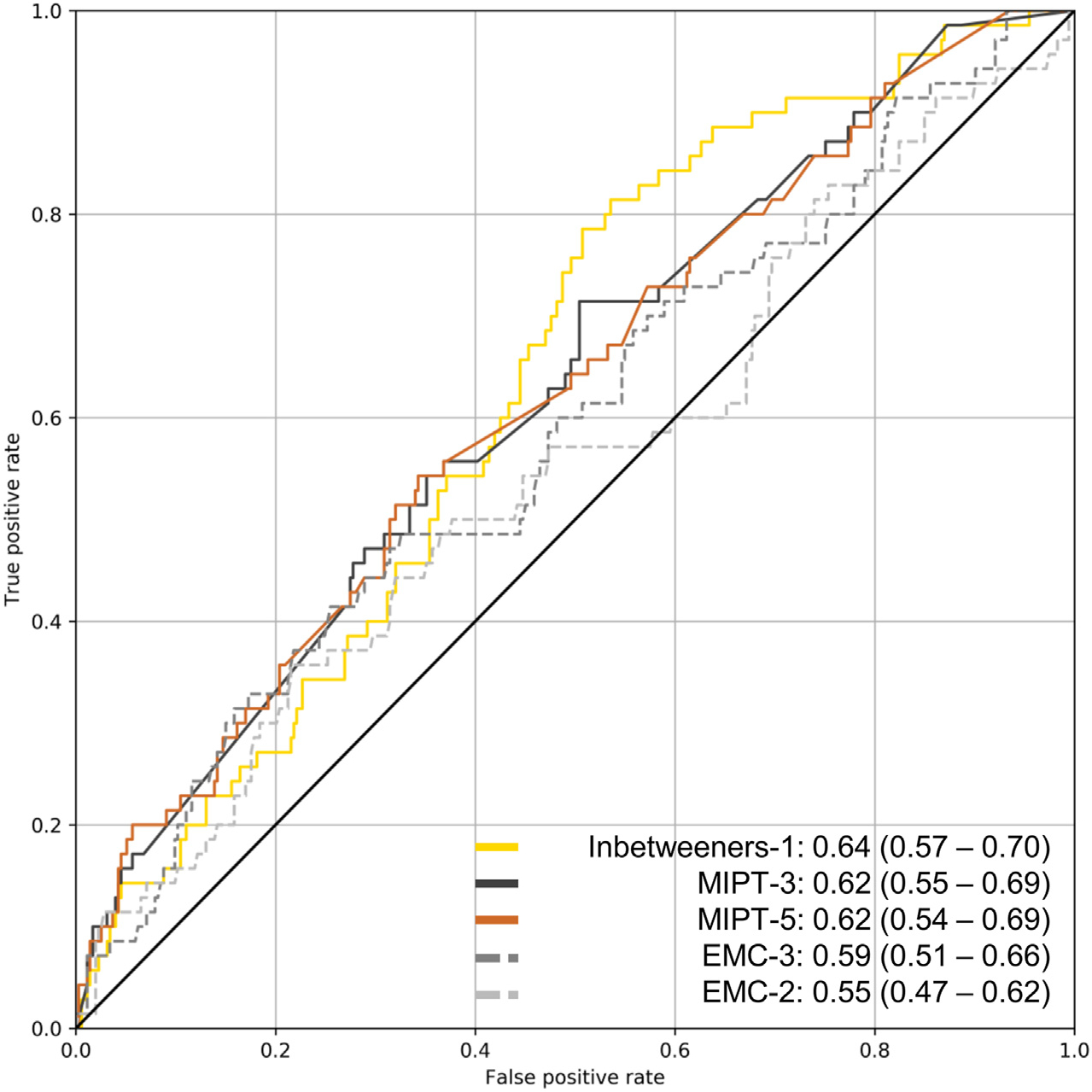
Receiver operating characteristic curves and respective area under the curve (ROC AUC) values for the three algorithms with the highest ROC AUC (*Inbetweeners-1*, *OuluMIPT-3*, and *OuluMIPT-5*) and for two reference models (*EMC-2* (age, BMI, and mild symptoms) and *EMC-3* (age, BMI, KL grade, and mild symptoms)).

The ROC AUCs of all submitted algorithms varied from 0.501 to 0.636 (Table II). The algorithm with the highest ROC AUC was *Inbetweeners-1* with an ROC AUC of 0.636 (95% CI: 0.571–0.699), which was statistically significantly higher (*P* < 0.05) than the ROC AUCs of the *EMC-1*, *EMC-2*, and *UC-MRI* reference models according to the DeLong’s test. Fig. 1 shows the ROC curves for the three algorithms with the highest ROC AUC (*Inbetweeners-1*, *OuluMIPT-3*, and *OuluMIPT-5*) and for two reference models (*EMC-2* and *EMC-3*).

The BACCs of all submitted algorithms varied from 0.479 to 0.587 (Table III). The algorithm with the highest BACC was *Oulu-MIPT-3* with a BACC of 0.587 (95% CI: 0.520–0.648). Of the reference models, *EMC-4* and *UC-MRI* had the highest BACCs with BACCs of 0.506 (95% CI: 0.477–0.542) and 0.506 (95% CI: 0.479–0.534), respectively.

**Fig. 2:**
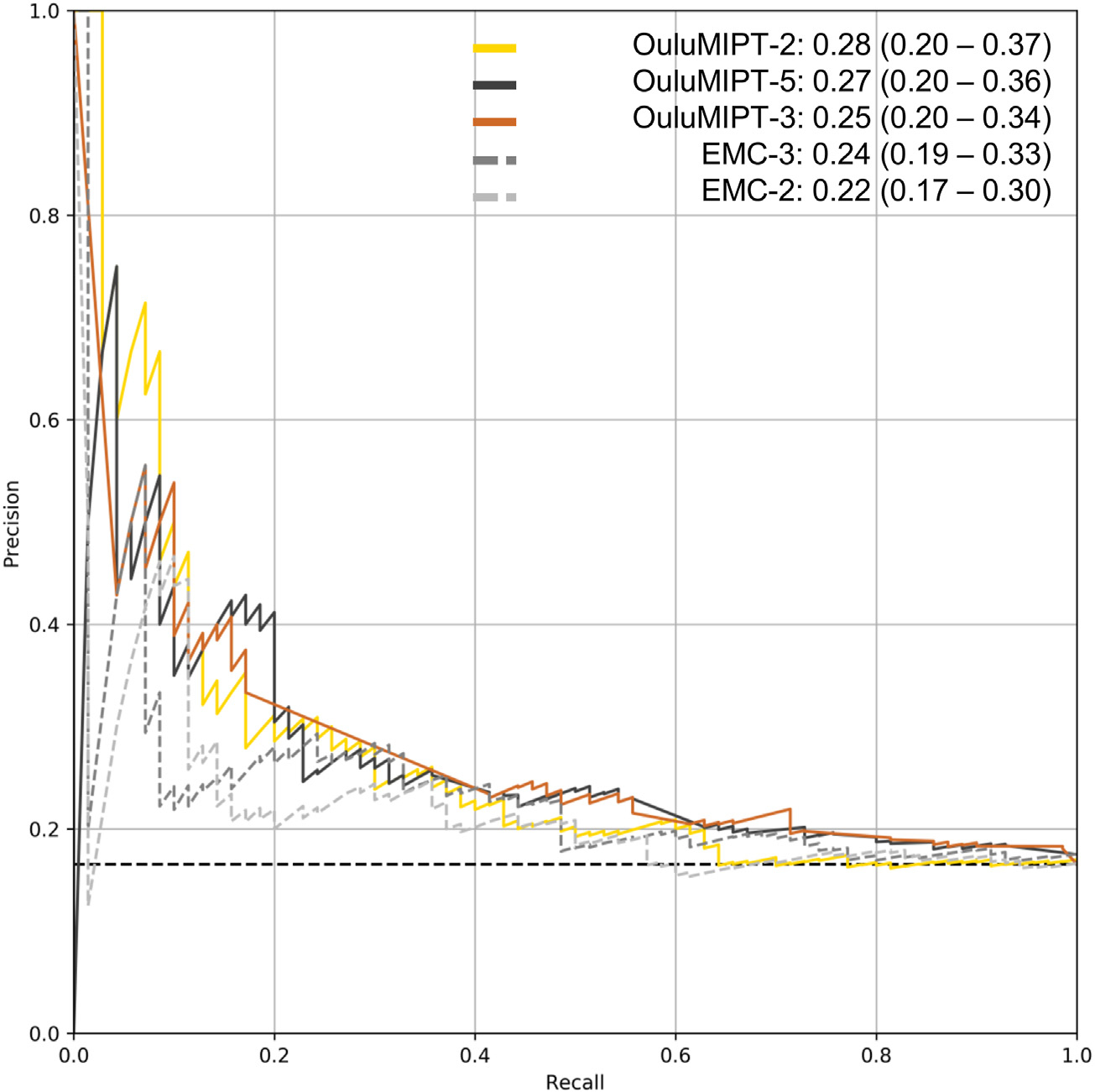
Precision-recall curves and respective area under the curve (PR AUC) values for the three algorithms with the highest PR AUC (*OuluMIPT-2*, *OuluMIPT-5*, and *OuluMIPT-3*) and for two reference models (*EMC-2* (age, BMI, and mild symptoms) and *EMC-3* (age, BMI, KL grade, and mild symptoms)).

The PR AUCs of all submitted algorithms varied from 0.167 to 0.276 (Table IV). The algorithm with the highest PR AUC was *OuluMIPT-2* with an PR AUC of 0.276 (95% CI: 0.199–0.367). Of the reference models, *EMC-3* had the highest PR AUC (0.244 (95% CI: 0.189–0.327)). Fig. 2 shows the PR curves for the three models with the highest PR AUC (*OuluMIPT-2*, *OuluMIPT-3,* and *OuluMIPT-5*) and for two reference models (*EMC-2* and *EMC-3*).

**Fig. 3:**
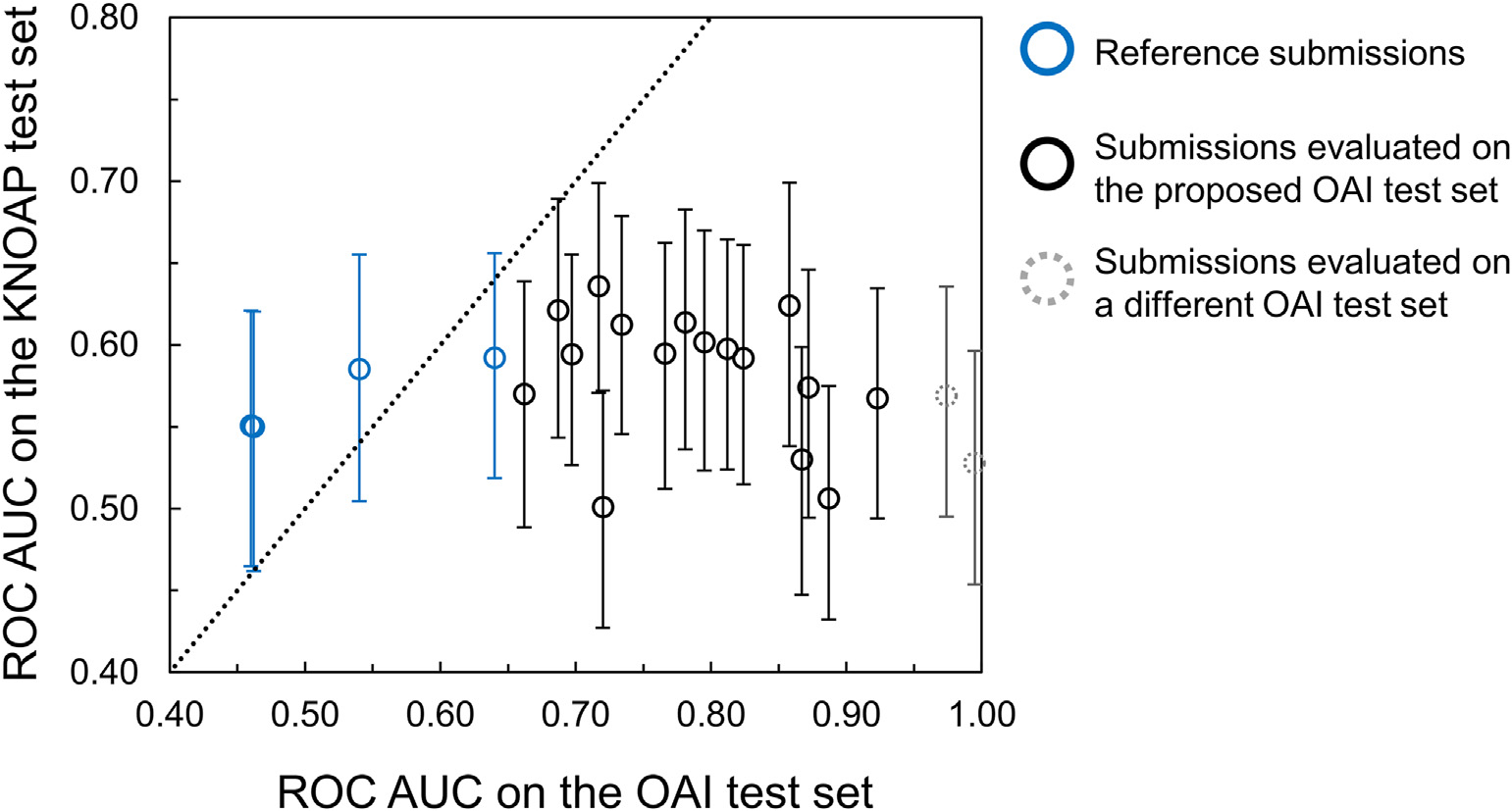
The relationship between the area under the receiver operating characteristic curve (ROC AUC) values of the algorithms on the KNOAP test set and on the OAI test set. Some submissions used a different OAI test set (dashed markers) than the proposed OAI test set for evaluating their models.

The majority of the algorithms had higher ROC AUC on the OAI test set than on the KNOAP test set (Fig. 3). It should be noted that some submissions used a different OAI test set than the proposed OAI test set for evaluating their models.

Post–challenge analysis showed varying sensitivities (from 0.00 to 0.757) and specificities (from 0.297 to 1.00) of the submitted algorithms (Supplementary Table 3). When one randomly selected knee per participant was used in the analyses, the absolute values of ROC AUC, BACC, and PR AUC were slightly higher than the original results, but the CIs were larger (Supplementary Tables 4, 5, and 6). Furthermore, *OuluMIPT-3* had the highest ROC AUC.

### X-ray image-based predictions

When looking at the submissions that used X-ray image data with or without clinical data, *Inbetweeners-1* had the highest ROC AUC (0.636 (95% CI: 0.571–0.699)). The algorithm with the highest BACC was *OuluMIPT-4* with a BACC of 0.579 (95% CI: 0.512–0.639). One model (*OuluMIPT-2*) used only X-ray image data (without covariate data) and had an ROC AUC of 0.570 (95% CI: 0.484–0.645) and a BACC of 0.547 (95% CI: 0.481–0.605).

### MRI-based predictions

There were two submissions that were based on MR images. One of those submissions (*CCF-MR*) had an ROC AUC of 0.612 (95% CI: 0.546–0.679) and a BACC of 0.553 (95% CI: 0.493–0.617). However, KL grade and varus malalignment are X-ray image-based variables and were included in the model and, therefore, the aforementioned submission is not purely MRI-based. The reference MRI submission (*UC-MRI*) had an ROC AUC of 0.537 (95% CI: 0.467–0.604) and a BACC of 0.506 (95% CI: 0.477–0.542).

## Discussion

In this work, we described the methodology and presented the results from the KNOAP2020 challenge. This is the first challenge organized on the prediction of knee OA incidence. A test set (MRI and X-ray image data along with clinical risk factors at baseline) with blinded ground truth was used to objectively compare different methods for prediction of incident symptomatic radiographic knee OA (combined ACR criteria) within 78 months. The model with the highest ROC AUC (0.64) used a CNN-based model to extract information from X-ray images and combined that information with clinical variables (i.e., age, BMI, and KL grade). The model with the highest BACC (0.59) ensembled three different models that used automatically extracted X-ray and MRI features along with clinical variables.

Previous studies have used various clinical risk factors for predicting the incidence of knee OA^5–8^. One study developed a logistic regression model using common risk factors for predicting incident symptomatic radiographic knee OA and reported an ROC AUC of 0.60 on the OAI data^5^. Another study used basic risk factors, genetic and biochemical markers, and radiographical scores and reported ROC AUCs of 0.75–0.86 for predicting incident radiographic knee OA in two external cohorts^6^. One study used a subset of OAI data and reported an ROC AUC of 0.72 for prediction of moderate/severe knee OA^8^. In another study, machine learning models with 112 and 10 predictors had ROC AUCs of 0.79 and 0.77 for prediction of incident radiographic knee OA^9^. The models included variables related to demographics, semi-quantitative MRI scores, cartilage T2 relaxation time values, symptoms, muscle strength, and physical activity. Lazzarini *et al.* (2017) used machine learning for prediction of incident symptomatic radiographic knee OA (ACR criteria) within 30-months in the PROOF study^7^. The model with the highest ROC AUC (0.79) included X-ray-based (baseline KL grade and shape modes), muscle strength, pain, and biochemical variables. Although the same dataset was used in this challenge, reasons for the better performance in the aforementioned study may include that they used the same dataset to train and test their models, availability of the outcome variable, shorter follow-up, and larger set of clinical variables.

Various deep learning methods have been used to predict the incidence and progression of knee OA. Tiulpin *et al.* (2019) predicted incidence and progression of radiographic knee OA using X-ray images and a modified ResNet model that was trained on the OAI dataset^14^. They reported ROC AUCs between 0.78 and 0.80 for prediction of the incidence and progression of OA on the MOST dataset using an image-based model and a model that combined image data and risk factors. Another study predicted the progression of radiographic medial joint space loss using a DenseNet and X-ray images from the OAI data and reported an ROC AUC of 0.86 for a model that combined image data and risk factors^12^. Leung *et al.* (2020) predicted the likelihood of a patient undergoing TKR using a case–control data from the OAI dataset^13^. They reported an ROC AUC of 0.87 for prediction of TKR surgery using X-ray images and a pre-trained ResNet. Tolpadi *et al.* (2020) predicted the occurrence of TKR within 5-years in the OAI dataset using a DenseNet^15^. They reported ROC AUCs of 0.83 and 0.89 for a model that combined MR images and risk factors and for a model that combined X-ray and risk factors, respectively. However, the MRI pipeline outperformed the X-ray pipeline for subjects without OA and with severe OA. Nguyen *et al.* (2021) predicted OA structural prognosis assessed by KL grade from X-ray and clinical variables and reported BACCs from 0.27 to 0.55^37^. In general, the performance of the models was lower in this study than in previous studies. However, direct comparison of the results is difficult due to differences in image datasets, data partitions, follow-up periods, evaluation metrics, and outcome variables. Furthermore, previous methods were not evaluated on a test set with blinded ground truth.

In this challenge, the model with the highest ROC AUC used a pre-trained ResNet34^13^ to extract information from X-ray images and combined this information with age, BMI, and KL grade to fit a logistic regression model. The model with the highest BACC used a Gaussian Naïve Bayesian model to ensemble three different models that used combinations of X-ray features (ResNet18 and Joint Shape-Joint Space features^38^), automatically extracted morphological cartilage features from sagittal MRI scans^39^ (segmented using deep learning^40^), and clinical variables. These results suggest that deep learning models pre-trained on a related task and an ensemble of the diverse models could be used to achieve higher performance for predicting incident knee OA.

Interestingly, the winning model did not use MRI data. However, there was a minor increase in ROC AUC values of some models after adding MRI data to the models. Due to the differences in the MRI data between the training and test sets, conclusions or recommendations on the use of MRI in prediction of the knee OA incidence are difficult to make. It should be also noted that the CIs were quite large and, therefore, the rankings should be interpreted with care. The finding that the final ranking depended on the metric is not surprising, as similar findings have been reported in previous challenges as well^19^. We chose ROC AUC and BACC as the main metrics because they have been widely used in previous literature and challenges^19,41,42^ and therefore are comparable to previous studies and because they are relatively easy to interpret. Due to the class imbalance in the test set, we also reported PR AUC values. The obtained PR AUC results indicate the difficulty in identification of knees that will develop OA within the follow-up.

For this challenge, we decided to split the PROOF dataset into a small training set and a large test set. The small training set was meant for fine-tuning. As the aim of this study was to predict the future incidence of knee OA, the applicability of the methods would be better if they would not need training or fine-tuning on the dataset where the prediction is made. Although the participants were free to use any data to train their methods, all except one submission used the OAI data for training. When comparing the results between the KNOAP test set and the OAI test set, better performance was seen on the OAI test set. One reason may be that the models were overfitted on the OAI training data. Another reason may be the difference between the training and test datasets, which can cause distribution shifts^43^. There might be some differences in the study populations as the OAI data was collected in the United States, whereas the test data was collected in the Netherlands. Imaging machines and image acquisition settings were also different between the datasets. For example, field strengths of the MRI scanners differed between the OAI and KNOAP test set. Although this challenge used a separate test dataset and the results thus provide insight how well the methods perform on unseen data, it should be noted that the test data consisted of overweight women aged between 50 and 62 years at baseline. As age and sex are known predictors of OA, inclusion of only women with relatively narrow age range could be one reason for lower performance compared to previous studies and it is unclear how the submitted models would generalize to other age groups and sex.

Many of the previous image analysis studies used structural outcome measure and did not include symptoms in their outcome variable. This may result in an inaccurate assessment of OA, as the presence of radiographic OA may be discordant with the presence of other structural findings and related symptoms^44,45^. We selected the ACR criteria because it is a long-used outcome and combines clinical features with radiography (‘clinical & radiographic ACR criteria’). We decided to use X-ray-based outcome as the availability of X-ray images and associated radiological scores is much greater than the availability of MRI data. In a future challenge, MRI data could be used as a reference standard provided that there are large enough datasets with labelled MRI available for model training. Furthermore, as the performance of all submitted models was limited in the test set demonstrating that the prediction of incident symptomatic radiographic knee OA is a complex problem, the impact of other input modalities and data (e.g., genetics) should be also investigated in the future.

This challenge has some limitations that need to be addressed. First, although the participants were allowed to use any data to train their methods, there is relatively limited data readily available for model training. This is because defining incident symptomatic radiographic OA requires baseline and follow-up clinical and imaging assessment that can be costly and difficult to obtain. Second, as we did not provide any precomputed features, segmentations of the MRI scans, or processed images, quite some effort was required from participants, which may have precluded some researchers from participating in the challenge. Third, the data contained both knees of most participants, which may have introduced some bias into the analysis.

In conclusion, the KNOAP2020 challenge established a benchmark for predicting incident symptomatic radiographic knee OA. This is the first challenge organized on the prediction of knee OA incidence. The performance of the submitted models on the independent test set with blinded ground truth was limited indicating that accurate prediction of incident symptomatic radiographic knee OA is a complex and still unsolved problem that requires additional investigation.

## Supplementary Material

Supplementary Material

## Figures and Tables

**Table I:** An overview of the submissions

Submission	Modality	Image feature extraction	Prediction model	Training data

*Akousist*	X-ray + MRI + clinical	X-ray: pre-trained ResNet-152; MRI: MRNet	XGBoost classifier	OAI (*n* = 3,654)
*CCF-Xray*	X-ray + clinical	X-ray: pre-trained VGG16	Logistic regression	OAI (*n* = 427)
*CCF-MR*	MRI + clinical	MRI: pre-trained AlexNet	Logistic regression	OAI (*n* = 427) + KNOAP train set (*n* = 30)
*Inbetweeners-1*	X-ray + clinical	X-ray: pre-trained Resnet34	Logistic regression	OAI (*n* = 1,581)
*Inbetweeners-2*	X-ray + clinical	X-ray: ResNet34 and ResNet50	Logistic regression	OAI (*n* = 1767)
*Inbetweeners-3*	X-ray + clinical	X-ray: ResNet34	Multi-layer perceptron	OAI (*n* = 1,581)
*Inbetweeners-4*	X-ray + clinical	X-ray: ResNet34	Multi-layer perceptron	OAI (*n* = 1,581)
*Inbetweeners-5*	X-ray + clinical	X-ray: pre-trained Resnet34	Logistic regression	OAI (*n* = 1,581)
*OuluMIPT-1*	X-ray + MRI + clinical	X-ray: joint shape and space (JS2) features; MRI: automatically extracted cartilage features	Gradient boosting machine	OAI (*n* = 432)
*OuluMIPT-2*	X-ray	X-ray: ResNet18	ResNet18	OAI (*n* = 432)
*OuluMIPT-3*	X-ray + MRI + clinical	X-ray: JS2 features and ResNet18; MRI: automatically extracted cartilage features	Ensemble of 3 models, Gaussian Naïve Bayesian	OAI (*n* = 432)
*OuluMIPT-4*	X-ray + clinical	X-ray: JS2 features	Gradient boosting machine	OAI (*n* = 432)
*OuluMIPT-5*	X-ray + MRI + clinical	X-ray: JS2 features and ResNet18; MRI: automatically extracted cartilage features	Ensemble of 3 models, Gaussian Naïve Bayesian	OAI (*n* = 432)
*TheRollingPebbles-0*	X-ray + clinical	X-ray: Pre-trained DenseNet121	Ensemble classifier	OAI (*n* = 3,654)
*TheRollingPebbles-1*	X-ray + MRI + clinical	X-ray: Pre-trained DenseNet121; MRI: Automatically extracted soft tissue and bone shape features	Ensemble classifier	OAI (*n* = 3,654)
*TheRollingPebbles-Filtered*	X-ray + MRI + clinical	X-ray: Pre-trained DenseNet121; MRI: Automatically extracted soft tissue and bone shape features	Ensemble classifier	OAI (*n* = 3,654)
*TheRollingPebbles-Full*	X-ray + MRI + clinical	X-ray: Pre-trained DenseNet121; MRI: Automatically extracted soft tissue and bone shape features	Ensemble classifier	OAI (*n* = 3,654)
*TheRollingPebbles-Ensemble*	X-ray + MRI + clinical	X-ray: Pre-trained DenseNet121; MRI: Automatically extracted soft tissue and bone shape features	Ensemble classifier	OAI (*n* = 3,654)
*UC-MRI′*	MRI	MRI: Automatically extracted cartilage and tibial bone features	Linear discriminant analysis	KNOAP train set (*n* = 30)
*EMC-1′*	Clinical	No image features	Logistic regression	OAI (*n* = 432)
*EMC-2′*	Clinical	No image features	Logistic regression	OAI (*n* = 432)
*EMC-3′*	Clinical	Manual	Logistic regression	OAI (*n* = 432)
*EMC-4′*	Clinical	Manual	Logistic regression	OAI (*n* = 432)

* Reference submission.

**Table II:** Area under the receiver operating characteristic curve (ROC AUC) values of the submissions

Rank	Submission	Modality	ROC AUC

1	*Inbetweeners-1*	X-ray + clinical	0.636 (0.571–0.699)
2	*OuluMIPT-3*	X-ray + MRI + clinical	0.624 (0.546–0.692)
3	*OuluMIPT-5*	X-ray + MRI + clinical	0.621 (0.539–0.690)
4	*Inbetweeners-5*	X-ray + clinical	0.614 (0.546–0.675)
5	*CCF-MR*	MRI + clinical	0.612 (0.546–0.679)
6	*OuluMIPT-4*	X-ray + clinical	0.602 (0.524–0.670)
7	*Inbetweeners-3*	X-ray + clinical	0.598 (0.524–0.665)
8	*CCF-Xray*	X-ray + clinical	0.595 (0.521–0.658)
9	*OuluMIPT-1*	X-ray + MRI + clinical	0.594 (0.512–0.662)
†	*EMC-4*	Clinical	0.592 (0.519–0.656)
10	*Akousist*	X-ray + MRI + clinical	0.592 (0.515–0.661)
†	*EMC-3*	Clinical	0.585 (0.505–0.655)
11	*TheRollingPebbles-Filtered*	X-ray + MRI + clinical	0.574 (0.505–0.637)
12	*OuluMIPT-2*	X-ray	0.570 (0.484–0.645)
13	*Inbetweeners-2*	X-ray + clinical	0.569 (0.495–0.636)
14	*Inbetweeners-4*	X-ray + clinical	0.567 (0.490–0.636)
†	*EMC-2*	Clinical	0.551 (0.465–0.621)*
†	*EMC-1*	Clinical	0.550 (0.462–0.620)*
†	*UC-MRI*	MRI	0.537 (0.467–0.604)*
15	*TheRollingPebbles-Full*	X-ray + MRI + clinical	0.530 (0.456–0.601)*
16	*TheRollingPebbles-Ensemble*	X-ray + MRI + clinical	0.528 (0.454–0.596)*
17	*TheRollingPebbles-0*	X-ray + clinical	0.506 (0.427–0.578)*
18	*TheRollingPebbles-1*	X-ray + MRI + clinical	0.501 (0.423–0.568)*

* Statistically significant difference (*P* < 0.05) between the submission and the first ranked submission according to the DeLong’s test.

† Reference submission.

**Table III:** Balanced accuracy (BACC) values of the submissions

Rank	Submission	Modality	BACC

1	*OuluMIPT-3*	X-ray + MRI + clinical	0.587 (0.520–0.648)
2	*OuluMIPT-4*	X-ray + clinical	0.579 (0.512–0.639)
3	*OuluMIPT-1*	X-ray + MRI + clinical	0.578 (0.506–0.639)
4	*CCF-Xray*	X-ray + clinical	0.571 (0.504–0.629)
5	*OuluMIPT-5*	X-ray + MRI + clinical	0.562 (0.501–0.616)
6	*TheRollingPebbles-Filtered*	X-ray + MRI + clinical	0.560 (0.494–0.615)
7	*CCF-MR*	MRI + clinical	0.553 (0.493–0.617)
8	*Akousist*	X-ray + MRI + clinical	0.550 (0.485–0.610)
9	*Inbetweeners-1*	X-ray + clinical	0.549 (0.507–0.592)
10	*OuluMIPT-2*	X-ray	0.547 (0.481–0.605)
11	*Inbetweeners-3*	X-ray + clinical	0.541 (0.489–0.592)
12	*Inbetweeners-5*	X-ray + clinical	0.531 (0.472–0.585)
13	*TheRollingPebbles-Full*	X-ray + MRI + clinical	0.527 (0.469–0.581)
14	*Inbetweeners-4*	X-ray + clinical	0.522 (0.493–0.553)
15	*TheRollingPebbles-Ensemble*	X-ray + MRI + clinical	0.515 (0.449–0.578)
16	*Inbetweeners-2*	X-ray + clinical	0.512 (0.490–0.539)
*	*UC-MRI*	MRI	0.506 (0.477–0.542)
*	*EMC-4*	Clinical	0.506 (0.479–0.534)
17	*TheRollingPebbles-1*	X-ray + MRI + clinical	0.504 (0.434–0.562)
*	*EMC-3*	Clinical	0.500 (0.500–0.500)
*	*EMC-2*	Clinical	0.500 (0.500–0.500)
*	*EMC-1*	Clinical	0.500 (0.500–0.500)
18	*TheRollingPebbles-0*	X-ray + clinical	0.479 (0.413–0.536)

* Reference submission.

**Table IV:** Area under the precision—recall curve (PR AUC) values of the submissions

Rank	Submission	Modality	PR AUC

1	*OuluMIPT-2*	X-ray	0.276 (0.199–0.367)
2	*OuluMIPT-5*	X-ray + MRI + clinical	0.271 (0.204–0.364)
3	*OuluMIPT-3*	X-ray + MRI + clinical	0.254 (0.196–0.342)
4	*Inbetweeners-1*	X-ray + clinical	0.245 (0.199–0.335)
*	*EMC-3*	Clinical	0.244 (0.189–0.327)
5	*CCF-Xray*	X-ray + clinical	0.239 (0.188–0.324)
6	*OuluMIPT-4*	X-ray + clinical	0.237 (0.187–0.321)
7	*CCF-MR*	MRI + clinical	0.237 (0.190–0.326)
8	*OuluMIPT-1*	X-ray + MRI + clinical	0.229 (0.179–0.310)
9	*Inbetweeners-5*	X-ray + clinical	0.227 (0.186–0.305)
10	*Inbetweeners-2*	X-ray + clinical	0.225 (0.179–0.309)
*	*EMC-4*	Clinical	0.224 (0.177–0.291)
*	*EMC-1*	Clinical	0.223 (0.173–0.308)
11	*Inbetweeners-3*	X-ray + clinical	0.222 (0.180–0.294)
*	*EMC-2*	Clinical	0.221 (0.172–0.303)
12	*Akousist*	X-ray + MRI + clinical	0.216 (0.177–0.290)
13	*Inbetweeners-4*	X-ray + clinical	0.210 (0.170–0.283)
14	*TheRollingPebbles-Filtered*	X-ray + MRI + clinical	0.198 (0.168–0.258)
15	*TheRollingPebbles-Ensemble*	X-ray + MRI + clinical	0.178 (0.149–0.234)
*	*UC-MRI*	MRI	0.177 (0.152–0.225)
16	*TheRollingPebbles-Full*	X-ray + MRI + clinical	0.175 (0.151–0.222)
17	*TheRollingPebbles-0*	X-ray + clinical	0.171 (0.146–0.219)
18	*TheRollingPebbles-1*	X-ray + MRI + clinical	0.167 (0.142–0.217)

* Reference submission.
