# Supplementary material for: The KNee OsteoArthritis Prediction (KNOAP2020) challenge: An image analysis challenge to predict incident symptomatic radiographic knee osteoarthritis from MRI and X-ray images
Source: Osteoarthritis Cartilage. Author manuscript; Available in PMC 2023 Jul 6. (PMC10323696; doi:10.1016/j.joca.2022.10.001)
Supplement: Supplementary Material [file NIHMS1905689-supplement-Supplementary_Material.docx]

**Supplementary Material to “The KNee OsteoArthritis Prediction (KNOAP2020) Challenge: An image analysis challenge to predict incident symptomatic radiographic knee osteoarthritis from MRI and X-ray images”**

**Supplementary Table 1.** Parameters of the magnetic resonance imaging protocol.

| Scanner | Field strength (T) | Sequence | TR (ms) | TE (ms) | Flip angle (degrees) | Slice thickness/  spacing (mm) | |
| --- | --- | --- | --- | --- | --- | --- | --- |
| Philips Intera | 1.0 | Coronal 2D PD | 1525 | 24 | 90 | 3/3.3 | |
| Philips Intera | 1.0 | Sagittal 3D WE | 22 | 11 | 25 | 3/1.5 | |
| Siemens Symphony | 1.5 | Coronal 2D PD | 1690 | 17 | 120 | 3/3.3 | |
| Siemens Symphony | 1.5 | Sagittal 3D WE | 2 | 6 | 25 | 1.5/1.5 | |
| Siemens Magnetom Essenza | 1.5 | Coronal 2D PD | 2500 | 27 | 150 | 3/3.6 | |
| Siemens Magnetom Essenza | 1.5 | Sagittal 3D WE | 21 | 8 | 25 | 1.5/1.5 | |
| PD = proton density, TE = time to echo, TR = repetition time, WE = water excitation | | | | | | |  |

**Supplementary Table 2.** Distribution of knees with and without incident symptomatic radiographic knee osteoarthritis (iSRKOA) between different scanners used to acquire the study data.

|  | KNOAP-train | |  | KNOAP-test | |
| --- | --- | --- | --- | --- | --- |
| Scanner | Controls (n=25) | iSRKOA (n=5) |  | Controls (n=353) | iSRKOA (n=70) |
| MRI: |  |  |  |  |  |
| Philips Intera | 10 (77%) | 3 (23%) |  | 143 (79%) | 37 (21%) |
| Siemens Symphony | 12 (86%) | 2 (14%) |  | 169 (84%) | 31 (16%) |
| Siemens Magnetom Essenza | 3 (100%) | 0 (0%) |  | 41 (95%) | 2 (5%) |
| X-ray: |  |  |  |  |  |
| General Electric | 15 (79%) | 4 (21%) |  | 231 (81%) | 54 (19%) |
| Swissray | 10 (91%) | 1 (9%) |  | 122 (88%) | 16 (12%) |

**Supplementary Table 3.** Post-challenge analysis of the sensitivity and specificity values of the submissions.

| **Submission** | **Sensitivity** | **Specificity** |
| --- | --- | --- |
| *Akousist* | 0.514 (0.386 – 0.629) | 0.586 (0.533 – 0.637) |
| *CCF-Xray* | 0.600 (0.486 – 0.714) | 0.541 (0.490 – 0.592) |
| *CCF-MR* | 0.486 (0.371 – 0.600) | 0.620 (0.569 – 0.671) |
| *Inbetweeners-1* | 0.143 (0.071 – 0.229) | 0.955 (0.932 – 0.975) |
| *Inbetweeners-2* | 0.043 (0.000 – 0.100) | 0.980 (0.966 – 0.992) |
| *Inbetweeners-3* | 0.243 (0.157 – 0.343) | 0.839 (0.793 – 0.875) |
| *Inbetweeners-4* | 0.071 (0.014 – 0.129) | 0.972 (0.952 – 0.986) |
| *Inbetweeners-5* | 0.286 (0.186 – 0.386) | 0.776 (0.734 – 0.819) |
| *OuluMIPT-1* | 0.586 (0.457 – 0.686) | 0.569 (0.516 – 0.623) |
| *OuluMIPT-2* | 0.629 (0.500 – 0.743) | 0.465 (0.411 – 0.513) |
| *OuluMIPT-3* | 0.457 (0.343 – 0.571) | 0.717 (0.666 – 0.762) |
| *OuluMIPT-4* | 0.657 (0.529 – 0.757) | 0.501 (0.450 – 0.552) |
| *OuluMIPT-5* | 0.314 (0.200 – 0.414) | 0.810 (0.768 – 0.847) |
| *TheRollingPebbles-0* | 0.643 (0.529 – 0.743) | 0.314 (0.263 – 0.363) |
| *TheRollingPebbles-1* | 0.514 (0.386 – 0.629) | 0.493 (0.439 – 0.541) |
| *TheRollingPebbles-Filtered* | 0.686 (0.571 – 0.786) | 0.433 (0.380 – 0.482) |
| *TheRollingPebbles-Full* | 0.757 (0.643 – 0.843) | 0.297 (0.246 – 0.346) |
| *TheRollingPebbles-Ensemble* | 0.529 (0.400 – 0.643) | 0.501 (0.448 – 0.552) |
| *UC-MRI**^*^* | 0.071 (0.014 – 0.143) | 0.941 (0.915 – 0.963) |
| *EMC-1^*^* | 0.000 (0.000 – 0.000) | 1.000 (1.000 – 1.000) |
| *EMC-2^*^* | 0.000 (0.000 – 0.000) | 1.000 (1.000 – 1.000) |
| *EMC-3^*^* | 0.000 (0.000 – 0.000) | 1.000 (1.000 – 1.000) |
| *EMC-4^*^* | 0.057 (0.014 – 0.114) | 0.955 (0.932 – 0.972) |

^*^Reference submission.

**Supplementary Table 4.** Post-challenge analysis of the area under the receiver operating characteristic curve (ROC AUC) values of the submissions when one randomly selected knee per participant was included in the analyses (*n* = 226).

| **Rank** | **Submission** | **Modality** | **ROC AUC** |
| --- | --- | --- | --- |
| 1 | *OuluMIPT-3* | X-ray + MRI + clinical | 0.667 (0.568 – 0.749) |
| 2 | *OuluMIPT-5* | X-ray + MRI + clinical | 0.664 (0.564 – 0.748) |
| 3 | *Inbetweeners-1* | X-ray + clinical | 0.660 (0.565 – 0.747) |
| 4 | *Inbetweeners-5* | X-ray + clinical | 0.642 (0.551 – 0.732) |
| 5 | *CCF-Xray* | X-ray + clinical | 0.631 (0.525 – 0.723) |
| 6 | *OuluMIPT-4* | X-ray + clinical | 0.630 (0.531 – 0.723) |
| 7 | *OuluMIPT-2* | X-ray | 0.617 (0.515 – 0.720) |
| 8 | *Inbetweeners-3* | X-ray + clinical | 0.616 (0.506 – 0.711) |
| * | *EMC-4* | Clinical | 0.611 (0.518 – 0.693) |
| 9 | *CCF-MR* | MRI + clinical | 0.609 (0.503 – 0.699) |
| 10 | *OuluMIPT-1* | X-ray + MRI + clinical | 0.603 (0.503 – 0.688) |
| * | *EMC-3* | Clinical | 0.602 (0.485 – 0.697) |
| 11 | *Inbetweeners-2* | X-ray + clinical | 0.595 (0.493 – 0.679) |
| 12 | *Akousist* | X-ray + MRI + clinical | 0.593 (0.497 – 0.685) |
| 13 | *Inbetweeners-4* | X-ray + clinical | 0.574 (0.461 – 0.669) |
| 14 | *TheRollingPebbles-Filtered* | X-ray + MRI + clinical | 0.574 (0.477 – 0.664) |
| * | *EMC-2* | Clinical | 0.558 (0.445 – 0.654) |
| * | *EMC-1* | Clinical | 0.554 (0.443 – 0.651)^3^ |
| 15 | *TheRollingPebbles-Full* | X-ray + MRI + clinical | 0.539 (0.439 – 0.632)^1,2,3^ |
| 16 | *TheRollingPebbles-Ensemble* | X-ray + MRI + clinical | 0.537 (0.436 – 0.629)^1,2,3^ |
| * | *UC-MRI* | MRI | 0.520 (0.419 – 0.613)^1,2,3^ |
| 17 | *TheRollingPebbles-0* | X-ray + clinical | 0.499 (0.403 – 0.586)^1,2,3,4,5,6^ |
| 18 | *TheRollingPebbles-1* | X-ray + MRI + clinical | 0.497 (0.399 – 0.584)^1,2,3,4,5,6^ |
| ^n^Statistically significant difference (*p*<0.05) between the submission and the n-th ranked submission according to the DeLong’s test. ^*^Reference submission. | | | |

**Supplementary Table 5.** Post-challenge analysis of the balanced accuracy (BACC) values of the submissions when one randomly selected knee per participant was included in the analyses (*n* = 226).

| **Rank** | **Submission** | **Modality** | **BACC** |
| --- | --- | --- | --- |
| 1 | *OuluMIPT-3* | X-ray + MRI + clinical | 0.624 (0.540 – 0.704) |
| 2 | *OuluMIPT-4* | X-ray + clinical | 0.615 (0.530 – 0.685) |
| 3 | *OuluMIPT-5* | X-ray + MRI + clinical | 0.601 (0.520 – 0.675) |
| 4 | *CCF-Xray* | X-ray + clinical | 0.592 (0.507 – 0.665) |
| 5 | *Inbetweeners-3* | X-ray + clinical | 0.586 (0.501 – 0.665) |
| 6 | *Inbetweeners-1* | X-ray + clinical | 0.578 (0.518 – 0.644) |
| 7 | *TheRollingPebbles-Filtered* | X-ray + MRI + clinical | 0.569 (0.481 – 0.645) |
| 8 | *OuluMIPT-2* | X-ray | 0.568 (0.489 – 0.644) |
| 9 | *Akousist* | X-ray + MRI + clinical | 0.567 (0.481 – 0.645) |
| 10 | *CCF-MR* | MRI + clinical | 0.562 (0.475 – 0.640) |
| 11 | *Inbetweeners-5* | X-ray + clinical | 0.550 (0.470 – 0.627) |
| 12 | *OuluMIPT-1* | X-ray + MRI + clinical | 0.548 (0.458 – 0.625) |
| 13 | *TheRollingPebbles-Ensemble* | X-ray + MRI + clinical | 0.540 (0.448 – 0.621) |
| 14 | *Inbetweeners-4* | X-ray + clinical | 0.537 (0.494 – 0.588) |
| 15 | *TheRollingPebbles-Full* | X-ray + MRI + clinical | 0.525 (0.451 – 0.590) |
| 16 | *Inbetweeners-2* | X-ray + clinical | 0.514 (0.484 – 0.552) |
| 17 | *TheRollingPebbles-1* | X-ray + MRI + clinical | 0.514 (0.430 – 0.599) |
| 18 | *TheRollingPebbles-0* | X-ray + clinical | 0.513 (0.434 – 0.584) |
| * | *EMC-4* | Clinical | 0.503 (0.459 – 0.548) |
| * | *UC-MRI* | MRI | 0.503 (0.460 – 0.548) |
| * | *EMC-3* | Clinical | 0.500 (0.500 – 0.500) |
| * | *EMC-2* | Clinical | 0.500 (0.500 – 0.500) |
| * | *EMC-1* | Clinical | 0.500 (0.500 – 0.500) |

^*^Reference submission.

**Supplementary Table 6.** Post-challenge analysis of the area under the precision-recall curve (PR AUC) values of the submissions when one randomly selected knee per participant was included in the analyses (*n* = 226).

| **Rank** | **Submission** | **Modality** | **PR AUC** |
| --- | --- | --- | --- |
| 1 | *OuluMIPT-2* | X-ray | 0.355 (0.247 – 0.489) |
| 2 | *OuluMIPT-5* | X-ray + MRI + clinical | 0.337 (0.240 – 0.480) |
| 3 | *Inbetweeners-1* | X-ray + clinical | 0.316 (0.234 – 0.450) |
| 4 | *OuluMIPT-3* | X-ray + MRI + clinical | 0.307 (0.230 – 0.431) |
| 5 | *CCF-Xray* | X-ray + clinical | 0.299 (0.217 – 0.428) |
| * | *EMC-3* | Clinical | 0.295 (0.205 – 0.435) |
| 6 | *Inbetweeners-5* | X-ray + clinical | 0.291 (0.214 – 0.432) |
| 7 | *OuluMIPT-4* | X-ray + clinical | 0.274 (0.208 – 0.398) |
| 8 | *CCF-MR* | MRI + clinical | 0.272 (0.203 – 0.390) |
| 9 | *Inbetweeners-3* | X-ray + clinical | 0.272 (0.203 – 0.395) |
| * | *EMC-2* | Clinical | 0.258 (0.180 – 0.381) |
| * | *EMC-1* | Clinical | 0.258 (0.180 – 0.378) |
| 10 | *OuluMIPT-1* | X-ray + MRI + clinical | 0.257 (0.187 – 0.365) |
| 11 | *Inbetweeners-2* | X-ray + clinical | 0.255 (0.191 – 0.368) |
| * | *EMC-4* | Clinical | 0.253 (0.192 – 0.348) |
| 12 | *Inbetweeners-4* | X-ray + clinical | 0.248 (0.187 – 0.367) |
| 13 | *Akousist* | X-ray + MRI + clinical | 0.243 (0.188 – 0.364) |
| 14 | *TheRollingPebbles-Filtered* | X-ray + MRI + clinical | 0.218 (0.174 – 0.313) |
| 15 | *TheRollingPebbles-Ensemble* | X-ray + MRI + clinical | 0.202 (0.161 – 0.292) |
| 16 | *TheRollingPebbles-Full* | X-ray + MRI + clinical | 0.197 (0.159 – 0.287) |
| * | *UC-MRI* | MRI | 0.186 (0.155 – 0.253) |
| 17 | *TheRollingPebbles-0* | X-ray + clinical | 0.173 (0.147 – 0.221) |
| 18 | *TheRollingPebbles-1* | X-ray + MRI + clinical | 0.169 (0.147 – 0.216) |

^*^Reference submission.

**Challenge entries**

The 23 algorithms submitted by seven teams are summarized below.

**Team:** Akousist

**Training data:** 3654 knees from the OAI.

**Features:** MRNet on coronal and sagittal MRI scans, pre-trained ResNet-152 for X-ray, all clinical variables.

**Missing data:** Missing values replaced with an average value of the variable.

**Prediction method:** XGBoost classifier.

**Team:** Cleveland Clinic (CCF)

**Training data:** 427 knees from the OAI (the matched OAI subset). For CCF-Xray model, 30 knees from the KNOAP finetune training data were also included in the training data. Data augmentation was used when training the CCF-MRI model. CCF-Xray model was trained using 5-fold cross-validation.

**Features:**

- CCF-Xray: pre-trained VGG-16 and all provided clinical variables.
- CCF-MRI: pre-trained AlexNet on coronal and sagittal MRI scans and all provided clinical variables.

**Missing data:** Missing values replaced with a constant value.

**Prediction method:** Logistic regression.

**Team:** Inbetweeners (NYULH)

**Training data:** 1581 – 1767 knees from the OAI (age and BMI matched with the test set). Inbetweeners-1 and Inbetweeners-5 models were trained using 7-fold cross-validation. Data augmentation was used when training the Inbetweeners-2, Inbetweeners-3, and Inbetweeners-4 models.

**Features:**

- Inbetweeners-1: ResNet-34 model trained to predict total knee replacement surgery from X-rays^1^, clinical variables (age, BMI, KL). The probability scores of the 5 models from the cross-validation were averaged.
- Inbetweeners-2: Both ImageNet pretrained and trained from scratch ResNet-34 and ResNet-50 models, clinical variables (age, BMI, KL, mild symptoms).
- Inbetweeners-3: ResNet-34 model for X-rays, clinical variables (age, BMI, and KL).
- Inbetweeners-4: Four ResNet-34 models for X-rays (1 with unsupervised data augmentation), clinical variables (age, BMI, and KL).
- Inbetweeners-5: Five ResNet-34 models trained to predict total knee replacement surgery from X-rays, clinical variables (age, BMI, and KL).

**Missing data:** No missing data.

**Prediction method:**

- Inbetweeners-1, Inbetweeners-2, and Inbetweeners-5: Logistic regression.
- Inbetweeners-3 and Inbetweeners-4: Multi-layer perceptron.

**Team:** OuluMIPT

**Training data:** 432 knees from the OAI (the matched OAI subset). The models were trained using 5-fold cross-validation.

**Features:**

- OuluMIPT-1: Joint shape and joint space features (JS2) representing the shape of the knee joint and the spacing of tibiofemoral joint extracted from X-rays^2^, automatically extracted 86 morphological cartilage features (based on volume, local thickness, and distance transform) from sagittal MRI scans^3^ (segmented using deep learning^4^), all covariates except varus malalignment, side of the knee (left/right), and all the features above for the contralateral knee (non-knee specific clinical variables were included only once).
- OuluMIPT-2: ResNet-18 model for X-rays.
- OuluMIPT-3: Ensemble of OuluMIPT-1, OuluMIPT-2, and OuluMIPT-4 models.
- OuluMIPT-4: Joint shape and joint space features (JS2) from X-rays, clinical (age, gender, BMI, and KL).
- OuluMIPT-5: Ensemble of OuluMIPT-1, OuluMIPT-2, and OuluMIPT-4 models.

**Missing data:** LightGBM default method. All records without input images were excluded.

**Prediction method:**

- OuluMIPT-1 and OuluMIPT-4: Gradient Boosting Machine to ensemble the 5 models from the cross-validation.
- OuluMIPT-2: ResNet-18.
- OuluMIPT-3 and OuluMIPT-5: Gaussian Naïve Bayesian with isotonic calibration.

**Team:** The Rolling Pebbles

**Training data:** All baseline knees from the OAI. Data augmentation was used when training the deep learning model for X-ray images.

**Features:**

- TheRollingPebbles-v0: DenseNet-121 pretrained to predict KL grades from X-ray images^5^. The used features included the predicted probability and the 12 features from the last output layer before the prediction layer.
- TheRollingPebbles-v1: DenseNet-121 pretrained to predict KL grades from X-ray images, automatically extracted soft tissue shape features (features from the last fully connected layer, logits, and probabilities) from sagittal MRI scans (segmented using deep learning), and automatically extracted bone shape features (features from the last fully connected layer, logits, and probabilities) from sagittal MRI scans (segmented using deep learning)^6^.
- TheRollingPebbles-vFiltered: as in TheRollingPebbles-v1, but a feature was filtered if its variance was lower than a pre-specified threshold (0.2) and *p*-value of the distribution of each feature between OAI and PROOF in the Kolmogorov-Smirnov two-sample test was less than 0.1.
- TheRollingPebbles-Full: as in TheRollingPebbles-v1, but a feature was filtered if its variance was lower than a pre-specified threshold (0.2).
- TheRollingPebbles-Ensemble: Ensemble model of TheRollingPebbles-v0, TheRollingPebbles-v1, TheRollingPebbles-vFiltered, and TheRollingPebbles-Full.

**Missing data:** Without or with multivariate imputation by chained equations using IterativeImputer in scikit-learn.

**Prediction method:**

- TheRollingPebbles-v0: Multiple candidate ensemble models, including balanced random forest, class weighted random forest, randomly under-sampled boost, and balanced bagging.
- TheRollingPebbles-v1: as in TheRollingPebbles-v0.
- TheRollingPebbles-vFiltered: as in TheRollingPebbles-v0.
- TheRollingPebbles-Full: as in TheRollingPebbles-v0.
- TheRollingPebbles-Ensemble: Ensemble model of TheRollingPebbles-v0, TheRollingPebbles-v1, TheRollingPebbles-vFiltered, and TheRollingPebbles-Full.

**Team:** UC-MRI

**Training data:** 30 knees from the KNOAP training dataset.

**Features:** Automatically extracted volume, thickness, contact area, congruity, homogeneity, cavity for cartilage compartments and tibial bone from sagittal MRI scans^7^.

**Missing data:** No missing data.

**Prediction method:** Linear Discriminant Analysis.

**Team**: EMC

**Training data**: 432 knees from the OAI training data (the matched OAI subset). The models were trained using 10-fold cross-validation.

**Features**:

- EMC-1: age, BMI.
- EMC-2: age, BMI, mild symptoms.
- EMC-3: age, BMI, baseline KL grade, mild symptoms.
- EMC-4: All available covariates.

**Missing data**: Missing values replaced with the most frequent value of the variable.

**Prediction method**: Logistic regression with L2 regularization.

**References**

1. Leung K, Zhang B, Tan J, Shen Y, Geras KJ, Babb JS, et al. Prediction of total knee replacement and diagnosis of osteoarthritis by using deep learning on knee radiographs: data from the osteoarthritis initiative. Radiology. 2020;296:584-93.

2. Bayramoglu N, Nieminen MT, Saarakkala S. A Lightweight CNN and Joint Shape-Joint Space (JS2) Descriptor for Radiological Osteoarthritis Detection. In: Medical Image Understanding and Analysis, Papież BW, Namburete AIL, Yaqub M, Noble JA Eds. Cham: Springer 2020:331-45.

3. Panfilov E, Tiulpin A, Nieminen MT, Saarakkala S, Casula V. Deep learning-based segmentation of knee MRI for fully automatic subregional morphological assessment of cartilage tissues: Data from the Osteoarthritis Initiative. J Orthop Res. 2021:1-12. <https://doi.org/0.1002/jor.25150>.

4. Panfilov E, Tiulpin A, Klein S, Nieminen MT, Saarakkala S. Improving Robustness of Deep Learning Based Knee MRI Segmentation: Mixup and Adversarial Domain Adaptation. 2019 IEEE/CVF International Conference on Computer Vision Workshop (ICCVW). 2019:450-9.

5. Norman B, Pedoia V, Noworolski A, Link TM, Majumdar S. Applying Densely Connected Convolutional Neural Networks for Staging Osteoarthritis Severity from Plain Radiographs. J Digit Imaging. 2019;32:471-7.

6. Morales Martinez A, Caliva F, Flament I, Liu F, Lee J, Cao P, et al. Learning osteoarthritis imaging biomarkers from bone surface spherical encoding. Magn Reson Med. 2020;84:2190-203.

7. Dam EB, Runhaar J, Bierma-Zienstra S, Karsdal M. Cartilage cavity-an MRI marker of cartilage lesions in knee OA with Data from CCBR, OAI, and PROOF. Magn Reson Med. 2018;80:1219-32.
